# Outbreak of Marek’s disease in a vaccinated broiler breeding flock during its peak egg-laying period in China

**DOI:** 10.1186/s12917-015-0493-7

**Published:** 2015-07-23

**Authors:** Xinyu Zhuang, Haitao Zou, Huoying Shi, Hongxia Shao, Jianqiang Ye, Ji Miao, Genghua Wu, Aijian Qin

**Affiliations:** Ministry of Education Key Laboratory for Avian Preventive Medicine, Yangzhou University, No. 12 East Wenhui Road, Yangzhou, Jiangsu 225009 P.R. China; Key Laboratory of Jiangsu Preventive Veterinary Medicine, Yangzhou University, Yangzhou, 225009 P.R. China; Jiangsu Co-innovation Center for the Prevention and Control of Important Animal Infectious Diseases and Zoonoses, Yangzhou, 225009 P.R. China

**Keywords:** Marek’s disease virus, Isolation, Gene analysis, Broiler breeding chicken, peak egg-laying period

## Abstract

**Background:**

Outbreaks of Marek’s disease (MD), caused by Marek’s disease virus (MDV), primarily occur in 10–12-week-old hens.

**Case presentation:**

We report a case of MD in a breeding flock of 24–30-week-old vaccinated broilers in China. The clinical signs in the affected chickens appeared at 24 weeks, and the incidence of tumours peaked at 30 weeks. The morbidity and mortality of the hens were 5 % and 80 %, respectively. Hematoxylin–eosin staining of the tissues showed the typical characteristics of MD. MDV infection was confirmed in the hens with an agar gel diffusion precipitation assay for the MD antigen in the feather follicle epithelium. An MDV strain, designated AH1410, was isolated from the blood lymphocytes. Sequence analyses of the *pp38*, *meq*, and *gB* genes revealed that strain AH1410 had molecular features consistent with a virulent, previously identified MDV.

**Conclusion:**

Our data provide evidence that not only is MDV becoming more virulent, but that the period of its onset in chickens is expanding. These findings provide the basis the molecular surveillance and further study of virulent MDV mutants and control strategies for MD in China.

## Background

Marek’s disease (MD) is caused by Marek’s disease virus (MDV), of the family *Herpesviridae*. MD was first reported in 1907 by Josef Marek and is characterised by T-cell lymphomas in the peripheral nerves or organs of 10–12-week-old hens [1]. MDV is currently distributed worldwide. Although vaccination plays a vital role in the prevention and control of MD globally, MDVs of increased virulence have been frequently isolated in some countries, even among vaccinated chickens, calling into question the current vaccination program against MDVs. The virulent MDVs (vMDVs) usually carry mutations in genes such as *pp38*, *meq*, or *gB*, that are associated with the oncogenicity and and their immune evasion [2, 3].

In China, a vaccine has been widely used to prevent MD in the poultry industry. However, the MD vaccine program has frequently failed, and vMDVs are occasionally isolated in vaccinated chickens [4]. In this study, we report a recent outbreak of MD in a vaccinated breeding broiler flock in China during its peak egg-laying period.

## Case presentation

### Clinical symptoms and pathological lesions

**Fig. 1:**
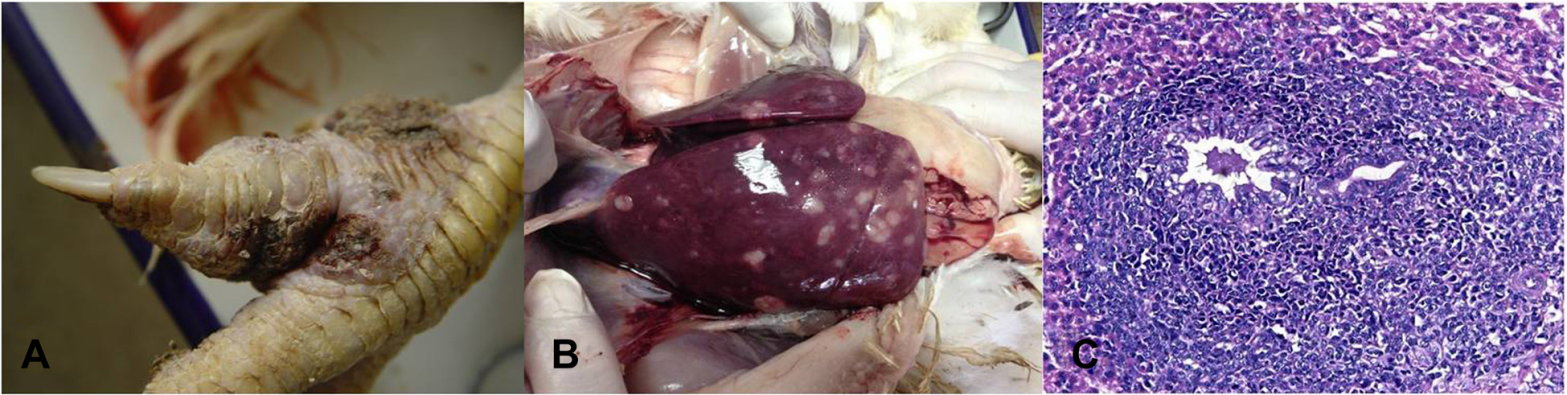
Clinical symptoms and pathological lesions. **a**: Cysts appeared in the toes; **b**: tumors appeared in the liver; **c**: histopathological tissue (HE, 400×)

The sick chickens were obtained from a large farm in Anhui Province, China. The owner of the chickens gave permission for their animals to be included in this study. The chickens were 24–30 weeks old. Tumours were found in their organs and skin. The occurrence of tumours increased progressively and reached its highest level (5 %) during the peak egg-laying period (30 weeks old), after which it began to decline. In the majority of sick chickens, the cysts first appeared between the toes (Fig. 1a) and then in the tarsometatarsus. All the chickens were hens. Tumours were observed in almost all tissues, particularly the liver (Fig. 1b). Immature infiltrated lymphocytes were observed in the liver and spleen during the histopathological diagnosis, ultimately destroying the tissue structure. Most of the darkly stained cells were lymphocytes, among which red eosinophilic granules were spotted. Many multinucleate cells with infiltrated lymphoid cells were present in the compartment of the liver (Fig. 1c), corresponding to the classical lesions of MDV.

### MDV antigen found in feathers

A band of MDV antigen was precipitated from the feather epithelia of the sick chickens. However, no band representing MDV antigen was found in the sera of the sick chickens. This suggests that the feather tips of the chickens excrete MDV particles. The anti-MDV antibody titres in the sera of the chickens were too low to detect with the agar gel diffusion precipitation (AGP) assay.

### Isolation and identification of MDV in the flock

**Fig. 2:**
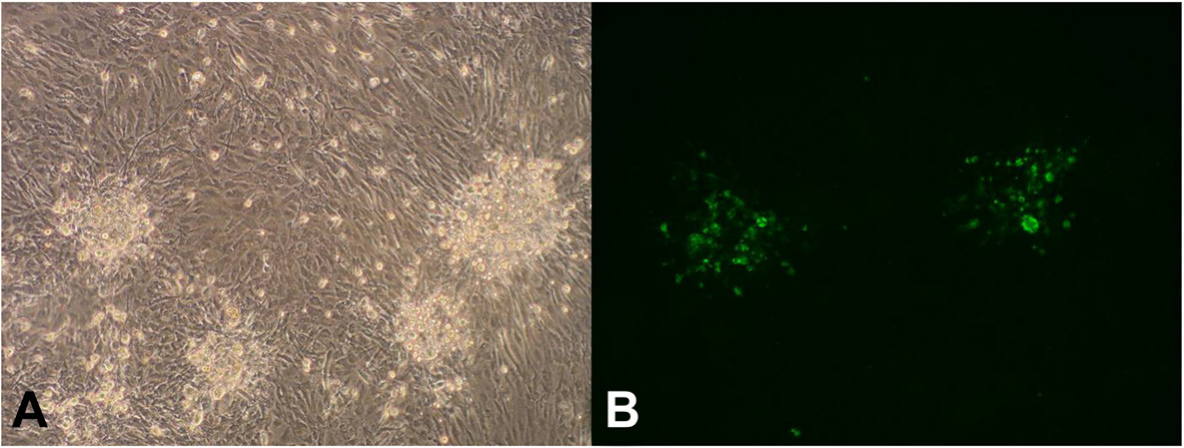
Viruses in CEF cells and indirect immunofluorescence assay with an MAb directed against MDV. **a**: MDV CPE in the CEF; **b**: immunofluorescence-positive cells tested with MAb BA4 against MDV-1

After two blind passages of chicken embryo fibroblast (CEF) cells, the presence of MDV (designated strain AH1410) in the CEF was confirmed when the typical cytopathic effect (CPE) appeared on the third day after infection (Fig. 2a). The infected cells became round and were highly refractive. When the cells were analysed with an anti-gB monoclonal antibody (Mab) BA4, specific uniform fluorescence was observed in both the nuclei and cytoplasm of the infected cells, but not in the CEF, suggesting that the cells showing the CPE reacted with the MDV-1 gB-specific Mab BA4 (Fig. 2b).

### MDV isolate has molecular features of vMDVs

**Fig. 3:**
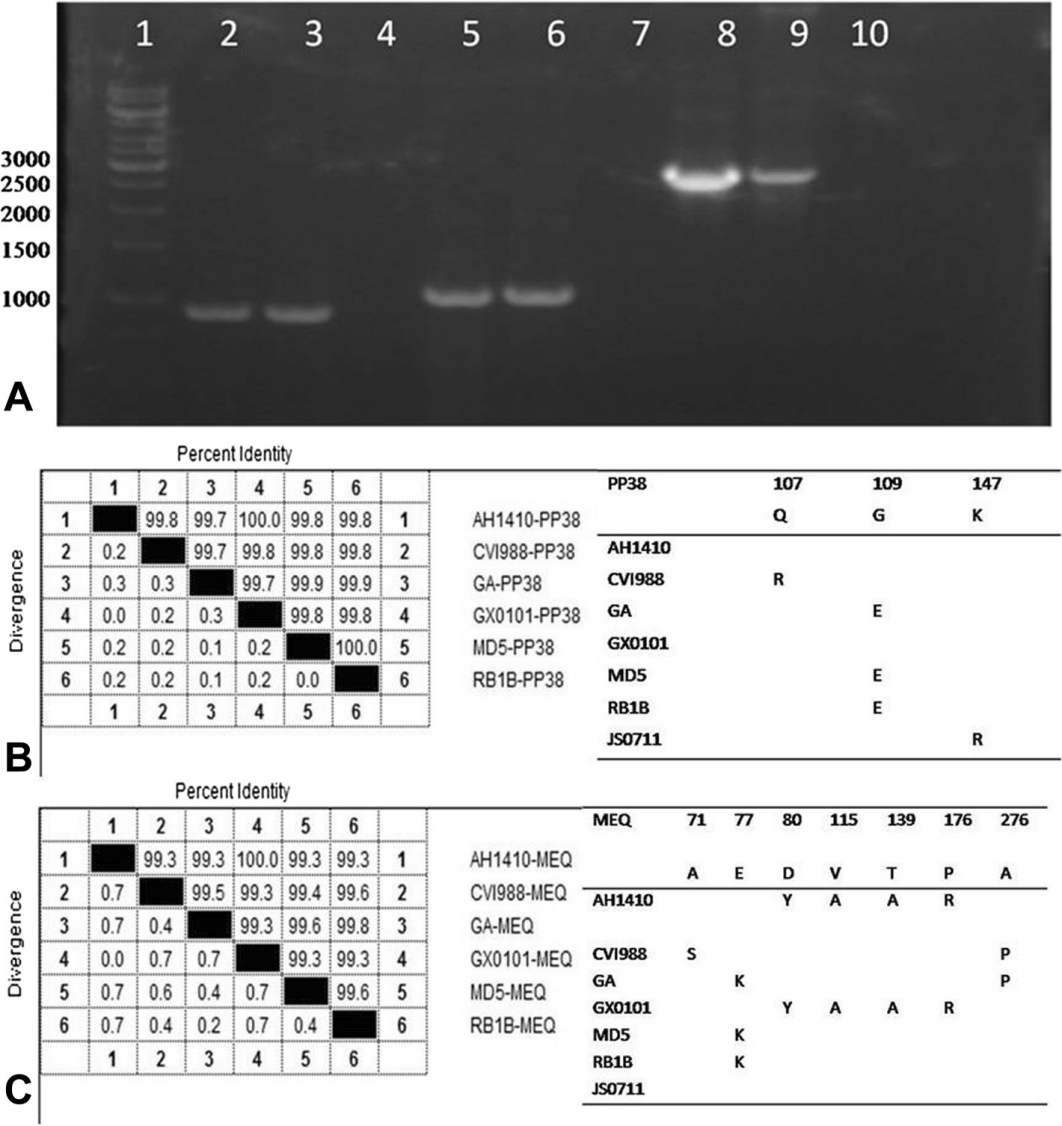
MDV genes amplified with PCR and the corresponding homology analyses. **a**: PCR results for the *meq*, *pp38*, and *gB* genes. Lane 1: 1-kb marker; lanes 4, 7, 10: negative controls; lanes 2 and 3: amplified *meq* gene; lanes 5 and 6: amplified *pp38* gene; lanes 8 and 9: amplified *gB* gene. **b**: Homology analysis and mutations of *pp38*. **c**: Homology analysis and mutations of *meq*

The genomic DNA was extracted from CEF infected with the isolated virus. The complete sequences of the *meq*, *gB*, and *pp38* genes were successfully amplified with PCR (Fig. 3a). A sequence analysis revealed that the *pp38* gene of AH1410 shared 99.3 %–100.0 % identity with those of reference strains, with only a single amino acid mutation at position 109 (E → G) in the deduced amino acid sequence (Fig. 3b). When we compared the *meq* sequences of reference strains published in the National Center for Biotechnology Information database with that of isolate AH1410, we found an insertion of 59 amino acids at position 194 of MEQ in the vaccine strain CVI988, so that its ORF encodes 398 amino acids. However, isolate AH1410 and other vMDVs all have ORFs encoding 339 amino acids in their *meq* genes, with 99.7 %–100.0 % homology. Only four amino acid mutations, at residues 80 (D → Y), 115 (V → A), 139 (T → A), and 176 (P → R), were found in isolate AH1410, which are identical to those in strain GX0101 (Fig. 3c). Analysis of the *gB* gene indicated that it was 100.0 % homologous between isolate AH1410 and other MDVs, with no mutations.

## Discussion

Marek’s disease is one of the most significant infectious diseases in poultry. In birds, it can result in tumours or immunosuppression, making the birds more sensitive to other pathogens. Although MD vaccines have been widely used in China for a long time, failures of MD vaccination occur frequently on some poultry farms. The improper selection of a vaccine could be the cause of vaccine failure [5]. Birds are also more susceptible to MDV mutants that have emerged in regions with more-virulent strains than to previous MDV isolates.

The *pp38* gene encodes a phosphoprotein of approximately 38 kDa that is necessary for both cell transformation and viral reactivation in the latent period [6, 7]. The *pp38* gene is conserved in all strains of MDV so far isolated. Previously, it was believed that *pp38* was not present in MDV strain CVI988 [8], but in recent years, the gene was shown to be expressed by CVl988, and a mutation at residue 107 (Q → R) is identified with Mab H19 [9, 10]. In this study, we found no mutation at residue 107 of the PP38 protein of the isolated strain AH1410. The *pp38* gene sequence was identical to that of the vMDVs. However, at residue 109 of pp38 in the vMDVs and the very virulent MDVs (vvMDVs), such as strains GA, MD5, and RB1B, glutamate is replaced by glycine, which differs from the sequence of strain AH1410, which is similar to that of other virulent viruses isolated in China, such as GX0101 and JS0711. Therefore, the isolated virus AH1410 is not the vaccine strain.

The MEQ protein, which is considered to be the major factor responsible for the occurrence of tumours, contains 339 amino acids [4]. However, the *meq* gene of CVI988 has an insertion of 177 bp compared with the genes of the vMDVs, which results in a 59-amino-acid insertion relative to the MEQ proteins of the vMDVs [11–13], that inhibits the expression of the *meq* gene [14]. Research has also indicated that mutations at positions 71 and 77 of the MEQ protein could be distinct in the vvMDVs [15]. Compared with CVI988, position 71 of strain AH1410 is mutated (S → A), as in the vMDVs, whereas position 77 is conserved. No deletion or insertion was found at position 194 and the ORF of AH1410 *meq* encodes 339 amino acids. We also found mutations at positions 80, 115, 139, and 176 of MEQ in our isolate AH1410, similar to those in GX0101 [16], suggesting that this type of strain has been circulating in China for many years.

With sequence alignments of the *meq*, *pp38*, and *gB* genes of strain AH1410 and other MDV isolates, we found that GX0101 and AH1410 are completely identical in these three genes. A previous study demonstrated that the virulence of GX0101 was at a level between those of GA and Md5 [16]. Collectively, these data indicate that the pathogenicity of isolate AH1410 may be similar to that of isolate GX0101.

The mutations and increasing virulence of MDV are thought to be responsible for the occurrence of MD on vaccinated farms. The failure of MD vaccination may be one reason for the outbreak reported here. Notably, the onset of MD in broilers generally starts at 4–6 weeks of age. In hens, MD usually occurs at 10–12 weeks of age [17]. However, in this case, the rate of tumours reached its highest level during the peak egg-laying period (30 weeks old).

Outbreaks among egg-laying chickens have also been reported in the mining area of Karnataka, India [18], and excessive losses from MD have recently been reported in adult laying flocks over the age of 40 weeks in Japan [19]. The data indicate that these birds may have contracted these infections later in life, with subsequent clinical signs. We also found that the feathers of the chickens were positive for the virus, whereas their sera were negative for MDV antigen, indicating that the chickens’ immune responses may have been suppressed in other ways, an unusual phenomenon and a disquieting trend. Another interesting observation is that all the diseased chickens were hens, which is consistent with an observation by Heier et al., who noted that males tended to be more resistant to MD than females [20].

It is necessary to strengthen the surveillance of circulating MDVs because new variants of virulent MDVs may appear at any time, and better vaccines against MD must be developed.

## Conclusion

Our data provide evidence that not only is MDV becoming more virulent, but that the period of its onset in chickens is also expanding. These findings provide the basis for the molecular surveillance of MDV, and further study of its virulence mutants and the control strategies required for MDV in China.

## Methods

### Chickens and samples

All the chickens were obtained from a breeding broiler chicken farm in Anhui, China. The chickens were immunized with the CVI988 vaccine at 1 day old. This vaccine had been used on the chicken farm for many years by skilled workers who vaccinate the chickens according to standard procedures. The clinical symptoms presented between 24 and 30 weeks. The morbidity and mortality of the hens were 5 % and approximately 80 %, respectively. Samples were obtained from the sick chickens. *Avian leukosis virus* and *Reticuloendotheliosis virus* infections were detected with PCR and virus isolation in our initial pathogen screening. The chickens reported here were negative for both viruses. All the experiments complied with institutional animal care guidelines and were approved by the University of Yangzhou Animal Care Committee.

### Histochemical assay

A histopathological assay and hematoxylin–eosin (HE) staining of the tissues were performed as previously described [21]. In brief, the livers and spleens of the sick chickens were fixed in 10 % neutral formalin, dehydrated in alcohol, and embedded in paraffin. They were then stained with HE and screened with light microscopy.

### AGP assay

An AGP test was performed on 1 % agar plates containing 8 % NaCl. In the AGP test, CEF cells infected with MDV strain RB1B were used as the positive control antigen, and preserved sera from the MDV-infected chickens were used as the positive control sera. The positive antigen (20 μl) was placed in the central well, and the same volumes of positive sera and samples were placed in the surrounding wells. The feather follicle epithelium was tested for the presence of MDV antigen. The plates were then placed in a humidified cabinet at 37 °C, and the precipitation lines were examined after 72 h.

### Virus isolation

Lymphocytes were separated with Lymphocyte Separation Medium (Tianjin Haoyang Biological Manufacture Co., Ltd) from the anticoagulant blood samples collected from the chickens. Primary CEF cultures were prepared from 10-day specific-pathogen-free embryos and inoculated in six-well plates with Dulbecco’s modified Eagle’s medium (DMEM) containing 5 % foetal bovine serum (FBS) in a total volume of 2 ml and incubated for 24 h. Then 60 μl of lymphocytes was added to the six-well plates that had been spread with the CEF. The plates were incubated at 37 °C in 5 % CO_2_. After 24 h, the DMEM was replaced with DMEM maintenance medium containing 1 % FBS. The plates were then incubated in a culture chamber for 6 days with 2–3 blind passages.

### Indirect immunofluorescence assay

The CEF with CPE were fixed with acetone: ethanol solution (3:2) for 10 min and washed once with PBS. After the fixed cells were blocked with 1 % BSA in PBS, they were incubated with the MDV-specific Mab BA4 [22] for 30 min at 37 °C. After three washes with PBS, the cells were incubated with fluorescein isothiocyanate (FITC)-conjugated goat anti-mouse antibody (Sigma-Aldrich, USA) for another 30 min. After three washes with PBS, the cells were examined under a fluorescence microscope.

### PCR and sequence analysis

The *meq*, *pp38*, and *gB* genes of the isolates were amplified with the primers listed in Table 1. The DNA extracted from CEF infected with the isolate was used as the template. In the PCR system, 50 μl of reaction volume was used, consisting of 5 μl of 10 × *LA Taq* PCR buffer, 2 μl each of the two primers, 0.5 μl of *LA Taq* DNA polymerase, 38.5 μl of ddH_2_O, and 2 μl of the DNA template. The PCR parameters for the *pp38* and *meq* genes were: 95 °C for 5 min, 30 cycles of 94 °C for 60 s, 55 °C for 60 s, and 72 °C for 60 s, and then 72 °C for 10 min. The PCR parameters for the *gB* gene were: 95 °C for 5 min, 30 cycles of 94 °C for 60 s, 55 °C for 60 s, and 72 °C for 2 min, and then 72 °C for 10 min. The PCR products were separated on a 1 % DNA gel and then sequenced by Genscript (Nanjing, China). All the sequences were aligned and analysed with DNAStar.

**Table 1:** Primers used to amplify MDV genes

Target	Name	Sequence	Length
pp38	pp38-F	5-AATGGATCCATGGAATTCGAAGCAGAAC-3	903 bp
	pp38-R	5-ATTGTCGACAACATCGGGTACGGCTAC-3	
meq	meq-F	5-CGCGAATTCTACAGGTGTAAAGAGATG-3	1058 bp
	meq-R	5-TAACTCGAGTGCTGAGAGTCACAATGC-3	
gB	gB-F	5-CAGTCGACTATGCACTATTTTAG-3	2.8 kb
	gB-R	5-CAGGAATTCACAAGGAAAGCATCG-3	

## Abbreviations

MD: Marek’s disease
MDV: Marek’s disease virus
AGP: agar gel diffusion precipitation
CEF: chicken embryo fibroblast
FBS: foetal bovine serum
DMEM: Dulbecco’s modified Eagle’s medium
CPE: cytopathic effect
BSA: bovine serum albumin
FITC: fluorescein isothiocyanate
PCR: polymerase chain reaction
